# The Influence of Social Comparison and Peer Group Size on Risky Decision-Making

**DOI:** 10.3389/fpsyg.2016.01232

**Published:** 2016-08-17

**Authors:** Dawei Wang, Liping Zhu, Phil Maguire, Yixin Liu, Kaiyuan Pang, Zhenying Li, Yixin Hu

**Affiliations:** ^1^School of Psychology, Shandong Normal UniversityJinan, China; ^2^Department of Computer Science, National University of IrelandMaynooth, Ireland; ^3^Department of Psychology, University of Pittsburgh, PittsburghPA, USA

**Keywords:** social reference points, risky decision making, peer group influence, social framing, social comparison

## Abstract

This study explores the influence of different social reference points and different comparison group sizes on risky decision-making. Participants were presented with a scenario describing an exam, and presented with the opportunity of making a risky decision in the context of different information provided about the performance of their peers. We found that behavior was influenced, not only by comparison with peers, but also by the size of the comparison group. Specifically, the larger the reference group, the more polarized the behavior it prompted. In situations describing social loss, participants were led to make riskier decisions after comparing themselves against larger groups, while in situations describing social gain, they become more risk averse. These results indicate that decision making is influenced both by social comparison and the number of people making up the social reference group.

## Introduction

Since ancient times, people have been concerned not only about their own income but also about the income of others. For example, in the Analects of Confucius, the renowned philosopher observes “People do not worry about poverty, but rather about the uneven distribution of wealth.” Employees usually care about their colleagues’ salaries, and they also pay attention to the results of their friends’ investments. Intuitively, it seems that social comparison plays an important role in allowing people to both learn about themselves and assess their social environment. Festinger’s (1954) social comparison theory captures this phenomenon, proposing that people continuously compare their own situation to that of their peers. The information gleaned from such comparisons is relied upon to evaluate their ability and status.

In recent years, the importance of social comparison in human decision making and judgment has come to the fore. Research has suggested that social comparison is an automatic process affecting cognition at the neurological level, with widespread implications for behavior (Dehaene et al., 1998; Fliessbach et al., 2007). Social comparison is frequently cited as an explanation for the distortion of asset prices, for example, that involving the 2015 Chinese stock market crash. Buunk and Gibbons (2007) have argued that strong focus should be placed on the cognitive manifestations of social comparison, such as those involved in decision making.

### Social Reference Points (SRP)

Existing research in this area proposes that social comparison can be defined in terms of a ‘social reference point (SRP),’ allowing it to be manipulated experimentally (Fox and Dayan, 2004; Haisley et al., 2008; Hill and Buss, 2010; Linde and Sonnemans, 2012). Kahneman and Tversky (1979) first proposed the concept of reference points in their prospect theory, regarding it as a basis for decision making in the face of uncertainty. Though a vast body of research has supported the significance of reference points (e.g., Kahneman and Tversky, 1979; Thaler and Johnson, 1990; Rabin, 2000), most studies have focused only on the decision makers themselves, and not their social environment. Given that real world situations are nearly always influenced by social factors, there may be some aspects of decision making which prospect theory alone can not accommodate.

For example, in the case where an individual investor is facing a loss of 3000 yuan on the stock market, they can choose either to sell immediately or continue to hold, the latter being a risky option. According to prospect theory’s individual reference point, decision makers are inclined to choose the risky option, seeking to minimize their loss. However, if the decision maker knows that a friend has lost 2000 yuan on the same investment, they may change their risk attitudes and exhibit risk-averse behavior. Thus, although treating the individual as the reference point highlights the practical gains and losses, it ignores the reality that decisions are usually carried out in social environments where social comparisons are likely to exert strong influence. The existence of SRPs has received both theoretical and empirical support (e.g., Tversky and Kahneman, 1991; Fox and Dayan, 2004; Hill and Buss, 2010; Linde and Sonnemans, 2012). Tversky and Kahneman (1991, pp. 1046–1047) argue that “the reference state usually corresponds to the decision maker’s current position, (but) it can also be influenced by aspirations, expectations, norms, and social comparisons.”

Previous studies examining the influence of SRPs on risky decision making have tended to focus on the realm of monetary decision making (stock investment, lottery, salary etc.; e.g., Fox and Dayan, 2004; Linde and Sonnemans, 2012; Lu et al., 2015). According to Xie and Lu (2014), these SRPs can be divided into three types: social gain, social loss, and social neutral. In general, people tend to view an upward comparison with others as a social loss, while viewing a downward comparison as a social gain. Different judgments and decisions are made based on such gains and losses. Social loss can threaten self-concept (e.g., Brickman and Bulman, 1977; Muller and Fayant, 2010; Pettit and Lount, 2010), triggering a series of mental behaviors with varied implications for decision making (Hill and Buss, 2010).

### Social Group Size

Despite these results, some researchers continue to hold the view that social comparison has little impact on risky decisions (e.g., Rohde and Rohde, 2011). This discrepancy may be due to differences in how decisions are presented to participants in these studies, suggesting that many confounded factors interact with each other during the process. Further efforts to control and isolate such factors will thus be valuable to the field.

One possible factor is the size of the peer group constituting the SRP, one which previous studies have barely touched on. The SRPs in existing studies are mostly constrained to one or several significant others (for example, best friends, roommates, colleagues, etc.), and rarely based on anonymous larger groups. However, in collective societies in particular, people are likely to adopt macroscopic social perspectives to acquire information and modify behavior, bringing better-than-average effects and worse-than-average effects to the fore (Moore and Small, 2007).

With large-scale social indexes becoming increasingly accessible in the information era, this factor can be expected to impose increasing influence on individuals’ judgments. When students take standardized tests, for example, they can not only compare their grades with their best friend, roommates (a few people), classmates (dozens of people), but also evaluate themselves against data based on the entire school and even all of the people who have taken the test (tens of thousands of people).

Existing studies support the idea that social group size influences the results of social comparisons (Buckingham and Alicke, 2002; Klein, 2003; Garcia et al., 2013). For example, Klein (2003) introduced a comparison involving a single other as well as a reported average from a group of others in a feedback task. The results showed that, under the condition of positive feedback, participants derived more information when compared against an average of many others. In contrast, they became more competitive and also more reluctant to pass on useful clues when compared against a single other. From a statistical point of view, comparisons involving larger SRP groups may be more influential because they often represent a larger and more stable data source, derived from a diversified, representative group (Klein, 2003). One of the major aims of the current study is to examine how the factor of SRP group size affects social comparison results and risky decision making.

### Emotion and Framing Effects

Finally, rather than limiting the data collection to the decision-making task itself, we also consider other variables that reflect on the underlying mental processes. So-called rational decision making is rarely limited to optimizing objective benefits. It is a dynamic multifaceted process, often affected by subjective feelings, something which is particularly pertinent in collective societies such as China. For example, previous studies have shown that SRPs influence risky behavior by modulating self-concept and emotion (Fox and Dayan, 2004; Förster et al., 2008; Hill and Buss, 2010; Diener, 2012; Diener et al., 2013).

Though a consensus has yet to emerge regarding the psychological feelings involved in confronting losses and gains, in general people feel positive emotion in the gain domain, and negative emotion in the loss domain (Kahneman and Tversky, 1984; Ordóñez et al., 2000; Fox and Dayan, 2004). In social contexts people tend to consider situations of social disadvantage as the loss, and situations of social advantage as the gain (Fox and Dayan, 2004). To enhance our data, we measured both the social framing and the general feelings of participants during the decision-making process.

### Experiment

For the following study we recruited 312 college students as participants, and chose the ecologically valid ‘examination’ as the theme of the scenario, replacing the traditional monetary theme. Within Chinese college society, test results are one of the most important sources of social comparison among students, who constantly compare their scores against others, and rely on relative ranking to assess their achievement. Because of the strong competition in the college system, exam grades play an important role in students’ lives. Accordingly, test score is an appropriate theme for presenting a risky decision.

In brief, the experiment involves a scenario where students’ exam performance is compared against either (a) a single student, (b) the average of the class, or (c) the average of the school. This comparison score is drawn from one of three reference point levels: either a low SRP (social gain), a middle SRP (social neutral), or a high SRP (social loss), denoted as L-SRP, M-SRP, and H-SRP, respectively.

Students must then make a decision about whether they would choose to answer an additional question, whose content is unknown. Answering this question correctly would add an additional 10 points, while answering it incorrectly would lose 10 points. The only information participants are given about this unknown question is that the percentage of correct answers is around 50%, hence establishing a potentially risky decision.

The experiment investigates the following hypotheses:

Hypothesis 1: SRPs can significantly affect choices in risky decision making. Specifically, participants presented with high SRPs are more likely to be risk-seeking compared to those given low SRPs.
Hypothesis 2: The impact of SRPs on risky decision making differs depending on the size of the comparison group. Specifically, the impact from the average of a large group will be greater than that from a single other.
Hypothesis 3: The impact of different SRPs is also reflected by emotional indicators. Specifically, the impact of high SRPs on emotions during the decision making process will be greater than that of low SRPs, and larger group sizes will also lead to a larger effect.

## Materials and Methods

### Participants

Three hundred and twelve college students were recruited from three equal-standing universities on the Chinese mainland using the cluster sampling method; the group included both liberal-arts and science students. The results from participants who did not finish the experimental tasks were eliminated as invalid samples. Ultimately, there were 274 valid participants, including 125 males and 149 females, and the average age was 20.78 years (*SD* = 1.35 years).

### Experimental Design

The study used a 3 (SRP level: H-SRP, M-SRP, L-SRP) × 3 (group size: single other, class, school) between-subjects design. The independent variables were the SRP level and the group size. The dependent variables included the percentage of participants choosing the risky option, the risk-seeking index, framing, and general feelings. Simultaneously, we collected data intended to control for confounding variables. The specific measurements are as follows:

#### Risky Choice

The participants made a *final choice* between the safe option and the risky option. We recorded the percentage falling into each of the two categories.

#### Risk-Seeking Index

Participants rated which option was more attractive on a 9-point scale (1 = safe option, 9 = risky option). A response closer to 9 indicates that the risky option is more attractive, while a response closer to 1 indicates that the safe option is more attractive. Thus, a higher score indicates a stronger tendency to take risks.

#### Framing

Considering the scenario, participants were asked to define their interpretation of the situation using a 9-point scale (1 = *I define it as a failure*, 9 = *I define it as a success*).

#### General Feelings

We used Fox and Dayan’s (2004) index for the measurement of general feelings. This index consists of four items: sorrow-joy, it’s wonderful-it’s a pity, contentment-discontentment, and disappointment-satisfaction. A 7-point scale was used to measure these items. A higher total average score indicates more positive feelings.

#### Controlling of Confounding Variables

We measured three further variables, namely participants’ risk propensity (“Generally speaking, do you tend to be risk averse or risk-seeking in your usual state?”—1 = risk averse, 7 = risk-seeking), the perceived importance of test scores to the student (“Is the score recorded in your academic records important to you?”—1 = very unimportant, 9 = very important), and students’ typical exam performance (“In your usual state, your test scores generally tend to be above …”).

### The Experimental Materials

The experiment involved nine scenarios presented via a questionnaire. In this questionnaire participants were instructed to imagine that they were involved in an important test organized by the college, and that the scores would be recorded in their archives (for Chinese students, the archives will follow them through future education and work stages, serving as a primary reference representing their abilities; hence the archive is extremely important for Chinese students).

The questionnaire presents as follows: participants are told that they have achieved 80 points on the required questions, and notified of an SRP achievement of 70, 80, or 90 points, reflecting the L-SRP, M-SRP, and H-SRP conditions. They are also informed whether the SRP reflects the score of a single student, a class average score (30 people), or a school average score (3000 people). Next, participants are presented with the prospect of an additional optional question, which represents a potentially risky decision. The content of this optional question is unknown to participants, described only as having a 50% correct rate. There are two options. The safe option is to submit the paper and stop the test with a final result of 80 points; the risky choice is to answer the additional question, with an objective equal probability of receiving either 70 or 90 points (see Appendix for full details).

### Experimental Procedure

The experiment was conducted in a psychological laboratory. All of the participants were volunteers. They were randomly assigned to one of the nine conditions. During the experiment, graduate students in the psychology department performed the roles of experimental assistants and interviewers. The specific process was as follows: the interviewers firstly presented the relevant instructions and the confidentiality principle. The participants were then allowed to read the materials in a quiet environment and asked to imagine that they were participating in an important test and making real choices. After the participants had understood the scenario, they were asked to compare the attractiveness of the two options and indicate their final choice, as well as defining their framing of the situation and evaluating their general feelings. Next, they completed the measurements of the potential confounding variables. Finally, the participants were thanked and debriefed.

## Results

The valid data of 274 participants were analyzed using SPSS 16.0. All of the participants who indicated a risk-seeking index below 5 (the middle point in the 9-point scale) preferred the safe option, whereas those who indicated a risk-seeking index above 5 tended to prefer the risky choice.

### Risky Choice

A Pearson Chi Square test revealed that SRP level affects the percentage of participants choosing the risky option under the condition of school comparison, χ^2^(2,88) = 12.261, *p* < 0.01. As shown in **Table 1**, participants given the H-SRP (85.7%) and M-SRP (71.4%) were more risk-seeking than those given the L-SRP (43.8%), χ^2^(1,60) = 11.324, *p* < 0.01 and χ^2^(1,60) = 4.659, *p* < 0.05. In the single other and class average conditions, there were no significant differences between SRP level and risky choice. Participants in the L-SRP/school comparison condition were marginally more risk averse (43.8%) than those in the L-SRP/class comparison condition (66.7%), χ^2^(1,68) = 3.609, *p* = 0.057.

**Table 1:** Percentage of participants taking risky choice and risk-seeking index (Mean).

SRP	*N*	Risky-choice (%)	Risk-seeking index
**Single other**			
L-SRP	30	63.3	5.93
M-SRP	29	55.2	5.34
H-SRP	27	66.7	6.18
**Class**			
L-SRP	36	66.7	6.28
M-SRP	31	61.3	5.13
H-SRP	33	78.8	6.69
**School**			
L-SRP	32	43.8	5.25
M-SRP	28	71.4	6.07
H-SRP	28	85.7	6.96

*L-SRP, low social reference point (70 points); M-SRP, middle social reference point (80 points); H-SRP, high social reference point (90 points)*.

### Risk-Seeking Index

Pearson’s correlation tests indicated that there was no significant correlation between risk-seeking index and the control variables, except for risk propensity and typical performance. We conducted a covariance analysis by taking SRP and group size as the independent variables, risk index as the dependent variable, and risk propensity and typical performance as the covariates. The results showed a significant effect of SRP level, *F*(2,273) = 5.629, *p* < 0.01, η^2^ = 0.041: participants in the H-SRP condition were more risk-seeking than those given the M-SRP or L-SRP. Further analysis of the nine different conditions found that in the case of school comparison participants’ risk-seeking indexes were significantly different between the H-SRP and L-SRP conditions, *t*(58) = -3.750, *p* < 0.001. Meanwhile, there was a marginally significant difference between the participants’ risk-seeking index in the L-SRP/class comparison and the L-SRP/school comparison, *t*(66) = 1.930, *p* = 0.058. Descriptive statistics of risky choice and risk-seeking index in the experimental conditions are presented in **Table 1**.

**FIGURE 1:**
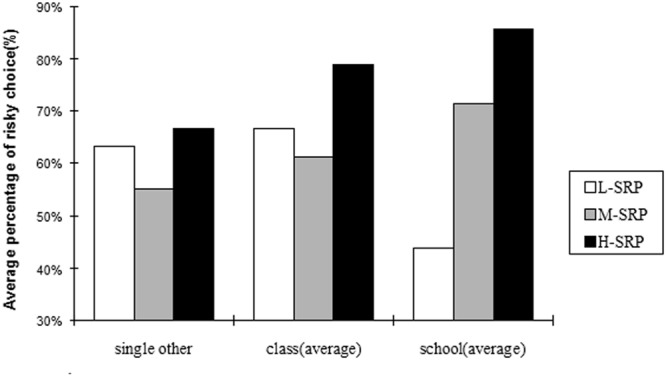
**Average percentage of risky choice in the experimental conditions**.

**Figure 1** shows the average percentage of the participants who chose the risky option across the nine conditions. The risky choice was chosen more often in the H-SRP condition than in the L-SRP and M-SRP conditions, a highly significant difference that is particularly salient in the case of school comparisons.

**FIGURE 2:**
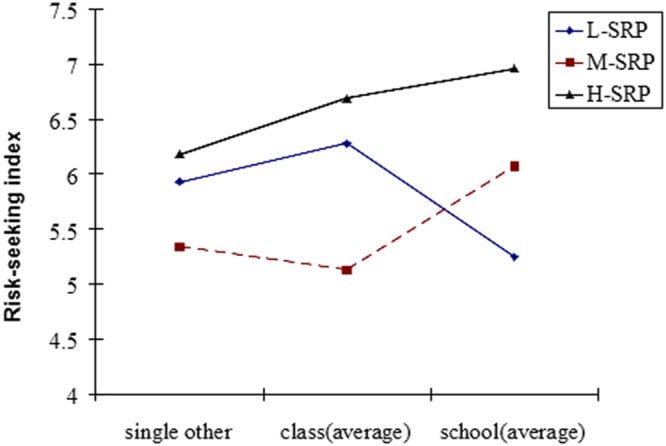
**Risk-seeking index in the experimental conditions**.

As **Figure 2** shows, in the L-SRP condition risk-seeking is reversed as comparison group size grows, especially to the school scale (3000 people), where it turns to risk aversion. However, in the H-SRP and M-SRP conditions, participants showed more risk-seeking behaviors as group size increased.

### Framing

**FIGURE 3:**
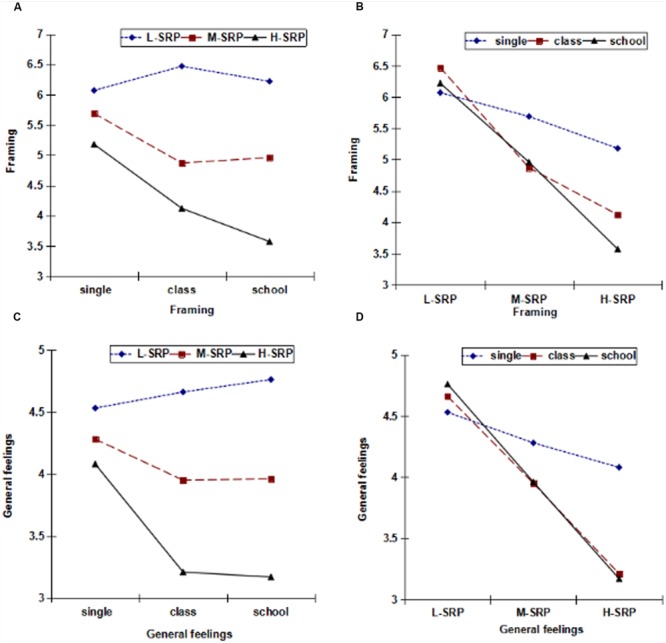
**Framing and General feelings in the experimental conditions**. **(A,C)** We analyzed group size with SRP level fixed. **(B,D)** We analyzed SRP level with group size fixed.

In general, participants tended to frame their situation as successful in the L-SRP condition, whereas they framed it as a failure in the H-SRP condition; this was true for all three group sizes. In the H-SRP condition the situation was more strongly framed as a failure for class and school comparisons than for comparisons involving a single other (see **Table 2**; **Figure 3**).

**Table 2:** Framing and General feelings (M ± SD).

SRP	*N*	Framing	General feelings
**Single other**			
L-SRP	30	6.07 ± 1.08	4.53 ± 0.68
M-SRP	29	5.69 ± 1.19	4.28 ± 0.73
H-SRP	27	5.18 ± 1.59	4.08 ± 1.04
**Class (average)**			
L-SRP	36	6.47 ± 1.34	4.66 ± 1.06
M-SRP	31	4.87 ± 1.75	3.95 ± 1.28
H-SRP	33	4.12 ± 1.71	3.21 ± 0.99
**School (average)**			
L-SRP	32	6.22 ± 1.24	4.76 ± 0.78
M-SRP	28	4.96 ± 1.77	3.96 ± 0.97
H-SRP	28	3.57 ± 1.28	3.17 ± 0.91

*L-SRP, low social reference point (70 points); M-SRP, middle social reference point (80 points); H-SRP, high social reference point (90 points).*

We conducted between-group ANOVAs by taking SRP level and group size as the independent variables, and framing as the dependent variable. This analysis yielded a main effect of group size, *F*(2,273) = 5.568, *p* < 0.01, η^2^ = 0.040, along with a main effect of SRP level, *F*(2,273) = 39.89, *p* < 0.001, η^2^ = 0.231. Simultaneously, the interaction between SRP level and group size was significant, *F*(4,273) = 3.65, *p* < 0.01, η^2^ = 0.052. For further simple effect analysis (see **Figure 3**) we fixed the SRP level and analyzed group size. In the H-SRP condition, the scenario was more likely to be defined as a failure for class comparisons and school comparisons than for comparisons involving a single other. There was no significant difference between school comparisons and class comparisons. In addition, we also analyzed SRP level with group size fixed. We found that for school comparisons, participants’ framing was significantly different between L-SRP and M-SRP, L-SRP and H-SRP, and M-SRP and H-SRP conditions. Specifically, participants framed their situation as more successful in the L-SRP condition, whereas more participants framed their situation as a failure in the H-SRP condition.

### General Feelings

We conducted several analyses of participants’ general feelings. The results showed that, for all three types of comparisons participants had more positive general feelings during the process of decision-making in the L-SRP condition. As might be expected, participants had relatively worse general feelings in the H-SRP condition. Moreover, participants had much worse feelings for the larger comparisons, especially in the case of school comparisons, than for comparisons involving a single other (see **Table 2**; **Figure 3**).

We also conducted between-group ANOVAs by taking SRP level and group size as the independent variables, and general feelings as the dependent variable. The analysis of general feelings yielded a main effect of group size, *F*(2,273) = 3.841, *p* < 0.05, η^2^ = 0.028, and a main effect of SRP level, *F*(2,273) = 31.837, *p* < 0.001, η^2^ = 0.194. Simultaneously, the interaction between SRP level and group size was significant, *F*(4,273) = 2.888, *p* < 0.05, η^2^ = 0.042. For further simple effect analysis (see **Figure 3**) we fixed the SRP level and analyzed group size. In the H-SRP condition we found a significant difference between single other and class comparisons, with participants’ general feelings being worse for class comparisons. A significant difference also existed between single other and school comparisons, with general feelings being worse for school comparisons. There was no significant difference between school comparisons and class comparisons. We also fixed group size and analyzed SRP level. **Figure 3D**, which graphs these results, reveals that, for comparisons involving single others, the change in feelings is relatively flat compared with larger group comparisons.

## Discussion

The results of the current study indicate that decision makers’ risky choices are affected by different SRPs. Generally, participants in the H-SRP condition were more risk-seeking than those in other conditions, with no significant differences between the M-SRP and L-SRP conditions. This result is generally consistent with our hypothesis that people take more risks when faced with social loss (Haisley et al., 2008). In addition, we observed that the impact of SRPs on risk-seeking behavior can be significantly manipulated by the size of the comparison group. In the case of single other and class comparisons, participants generally tended to be risk-seeking under L-SRP, in line with the findings of Lu et al. (2015). However, this risk-seeking inclination dropped dramatically in the case of school comparisons, with risky behavior reversed under L-SRP. This group was the most risk-averse one, while H-SRP/school comparison was the most risk-seeking one.

In summary, our results show that situations involving social loss (e.g., L-SRP) have a strong, consistent impact on individuals’ risky decision making, as evidenced by increased risk-seeking, while the effect of social gain (e.g., H-SRP) is more flexible, varying with group size. Furthermore, comparisons which are based on the average of large groups seem to impose greater influence on risky decisions, leading to greater divergence between conditions of social gain and social loss.

Our collection of data on framing and emotional response allows the results to be explored from the perspective of self-concept. Consistent with the findings of Fox and Dayan (2004), social losses led to significantly worse self-concept and general feelings than social gains. Although H-SRP (social loss) always had a greater negative impact on general feelings and self-concept than M-SRP and L-SRP (social neutral; social gain), increasing the size of the comparison group served to amplify that effect.

Only in the social loss condition, where self-concept was seriously threatened, did behavioral results evidence a consistent risk-seeking tendency. This finding is consistent with the idea that when an individual’s self-concept is threatened, they will actively seek repairing measures to maintain their positive self-concept. While both conservatism and risk-taking can serve to support the maintenance of self-concept (Zhang, 2009), the current study suggests that self-concept repairing measures tend to favor risk-taking. It has been shown that a negative stimulus, such as failure feedback, is more likely to attract attention (Rozin and Royzman, 2001; Peng and Zhou, 2005; Dyck et al., 2009). Individuals might thus be more easily influenced by negative information when under a situation of social loss, causing stronger negative feelings and the appearance of loss aversion behaviors.

Whereas the idea of threatened self-concept can explain attitudes for social loss, an alternative perspective is needed for understanding attitudes involving social gain. We suggest that these results can be understood in terms of opportunity-threat perception. Research has shown that people’s perceptions of opportunity and threat are different, and that such perceptions can be easily manipulated (e.g., Highhouse and Yüce, 1996; Xie and Wang, 2002). People view risky situations as an opportunity when positive results are expected, adopting a positive attitude toward the situation. In contrast, if negative results are more likely, individuals adopt a negative perception toward the situation and regard it as a threat. Different perceptions of risky situations may lead people to demonstrate different behaviors. For example, decision makers who are primarily concerned about threats will choose risk-averse options, while those who are attracted to opportunities will engage in risk-taking behaviors.

This subtle distinction between opportunity and threat seems vulnerable to social influence, and could be easily biased unconsciously in a social context where people take their cues from the gains and losses of others. Fox and Dayan (2004) argue that when people suffer from heavy losses and learn that others also did, they are more likely to regard the situation as threatening, and thus adopt a risk-averse attitude. It seems possible that our participants adopted a similar outlook when informed that many others had performed badly in the test, leading to a negative perception of the risky situation. However, our results also suggest that this effect is subtle enough that it is only manifested for comparisons involving a larger peer group.

Given that the experiment was carried out in mainland China, our results may also reflect cultural factors. Sedikides et al. (2003) argue that in countries with a collectivist culture, where humility is particularly emphasized (e.g., China, Japan), individuals are less self-expressive, especially after achieving decisive success. Yang and Gao (1991) suggest that Chinese self-evaluation is more negative because of the emphasizing of “introspection,” which drives people to engage in constant self-criticism. This introspection leads people to subscribe the doctrine of the golden mean: confident but not proud, modest but not inferior.

From this perspective we would expect Chinese students to be risk-averse when obviously successful, while becoming risk-seeking to counteract self-criticism and introspection when failing tasks. A different pattern of results to the one in this study might be found for individualist cultures, which emphasize the pursuit of extreme individual success.

A final noteworthy point is that, similar to previous studies, we also presented a social comparison involving a single other. However, in our study, this set of conditions did not demonstrate any strong effects. In sum, it seems that the effect on behavior and feelings for such comparisons is much more marginal than those based on the average of larger extended groups. As noted by Loewenstein et al. (1989), in order for comparisons involving single others to exert influence, some pre-existing interpersonal relationships may be required, a factor which was absent from our study.

### Limitations and Future Directions

Though the study provides interesting results, several limitations should be acknowledged, which set up directions for further research.

First, with respect to the experimental materials, the strategy we used and data sources we sampled are novel and have no precedent in the literature. This limits the potential for our data to be compared against other experiments. The issue, however, can be resolved by future investigations.

Second, people may employ different cognitive evaluation systems in different situations, such as those involving financial matters, academic performance, health etc. Accordingly, the experimental paradigm we used here should be applied to different decision-making contexts, providing a more diversified pool of results. The interaction between decision making and framing/general feelings should also be studied in more detail, and in different situations, to provide feedback on the stability and reliability of our results.

Finally, the greatest source of uncertainty concerning this study is the question of how greatly the results have been influenced by cultural background. Chinese culture combines influences from collectivist society and ingrained Confucianism, and plays a large role in organizing individuals’ thoughts and behaviors within Chinese society. In light of this, it would be valuable to conduct a counterpart experiment involving participants drawn from an individualistic cultural background. Heine et al. (2001) argue that individuals raised in an individualistic culture are more competitive, especially when they are close to the top. This contrasts markedly with individuals from collectivist cultures, who are reluctant to stand out. We can not know if the interesting reverse in risk-seeking for the L-SRP/school comparison condition stems from this reluctance, or whether it holds up across cultures.

## Conclusion

In this study we investigated the impact of SRPs from a novel perspective, namely the size of the group making up the reference point. As well as corroborating the finding that individuals are more risk-seeking in situations involving social loss, we have added to the converging evidence that group size also plays an important role. Specifically, we found that the larger the size of the comparison group, the greater the shift toward risk-seeking for social loss situations. In contrast, for situations involving social gain, the larger the size of the group, the greater the shift toward risk aversion. And the emotion generated in the decision process also plays an important role that influenced the final decision.

## Author Contributions

YH: Overall direction of the article. DW: Writing and direction of the article. LZ: Data collection and writing. PM: Direction of the article and English editing. KP: English editing. YL: Experimental implementation. ZL: Data analysis.

## Conflict of Interest Statement

The authors declare that the research was conducted in the absence of any commercial or financial relationships that could be construed as a potential conflict of interest.
