# Supplementary material for: The Influence of Social Comparison and Peer Group Size on Risky Decision-Making
Source: Front Psychol. 2016 Aug 17;7:1232. doi: 10.3389/fpsyg.2016.01232 (PMC4987381; doi:10.3389/fpsyg.2016.01232)
Supplement: Supplementary file 1 [file Data_Sheet_1.DOCX]

**Appendix**

*The example below is based on the high social reference point (90 points) and a class comparison (30 people).*

The school is now conducting a test in which all students must participate. The difficulty level of the question is appropriate and the maximum achievable score based on the required questions is 100 points. There is an additional, optional question for which participants can receive 10 points for a correct answer and lose 10 points for an incorrect answer. The test is conducted on a computer and the system will provide timely feedback.

The school arranges the test by administering it to each class as a unit. There is a delay and you are the last student in your class to take the test; you can see the average score among the other students (30 people) in your class. The system shows that your test score is 80 points when you complete the required questions and your class’s average score (the average score of all of the students after submitting the paper) is 90 points. You can choose to submit the paper to end the test. Alternatively, you can continue to answer the additional question, which may pose risks. If you choose to continue, there is a 50% probability of answering the additional question correctly and adding 10 points to your original score; however, there is also a 50% probability of answering the additional question incorrectly and losing 10 points from your original score. Your final score will be recorded in your archives.

In other words, you have two options:

Option A: submit the paper to end the test with a final score of 80 points; or

Option B: continue to answer, with an equal probability of a score of either 70 points or 90 points.

The average score (all of the students after submitting the paper) in your class is 90 points.
